# Imaging Correlation of Color Doppler Ultrasound, CT Angiography, and MR Angiography Findings in Extracranial Carotid and Vertebral Artery Disease Among Ischemic Stroke Patients: A Prospective Observational Study

**DOI:** 10.7759/cureus.116627

**Published:** 2026-09-21

**Authors:** Rashmi Singh, Mansi Patel, Kiran Katara, Sangeetha Panneerselvam, Anvitha Rose George, Nidhi Sai Sunil

**Affiliations:** 1 Department of General Medicine, Medical and Surgical Services, Amrita Hospital, Faridabad, IND; 2 Department of General Medicine, Dr. N. D. Desai Medical College &amp; Hospital, Nadiad, IND; 3 Department of General Medicine, Smt. B. K. Shah Medical Institute & Research Centre, Sumandeep Vidyapeeth (Deemed to be University), Vadodara, IND; 4 Department of General Medicine, Meenakshi Medical College Hospital and Research Institute, Kanchipuram, IND; 5 Department of General Medicine, Manipal Hospital Yelahanka, Bengaluru, IND; 6 Department of Respiratory Medicine, K S Hegde Medical Academy, Nitte University, Mangaluru, IND

**Keywords:** carotid plaque, carotid stenosis, color doppler ultrasound, ct angiography, extracranial carotid artery, ischemic stroke, mr angiography, plaque morphology, vascular imaging, vertebral artery

## Abstract

Background and objective

Extracranial carotid and vertebral artery (VA) atherosclerosis is an important contributor to ischemic stroke and recurrent cerebrovascular events. Color Doppler ultrasound is widely used as an accessible first-line modality, whereas CT angiography (CTA) and MR angiography (MRA) provide additional anatomical information regarding stenosis, plaque morphology, occlusion, and vascular variants. The objective of this study was to describe extracranial carotid and VA abnormalities in ischemic stroke patients using color Doppler ultrasound, CTA, and MRA and to assess aggregate distributional patterns of plaque characteristics and stenosis grades across these imaging modalities.

Methods

This prospective observational imaging correlation study included 50 patients with clinically suspected and neuroimaging-confirmed ischemic stroke who underwent extracranial vascular assessment. Demographic profile, vascular risk factors, Doppler plaque type, spectral broadening, CTA plaque morphology, MRA findings, and stenosis grades were analyzed. Additional descriptive and exploratory aggregate statistics included 95% CIs, chi-square tests for categorical distributions, aggregate modality-wise comparison of stenosis grades, and correlation of matched vessel-category stenosis percentages.

Results

The mean age was 63.6 ± 13.34 years (95% CI, 59.8-67.4), and 30 patients (60.0%) were male. Hypertension and smoking were the most frequent risk factors, each present in 27 patients (54.0%, 95% CI, 40.4-67.0), followed by diabetes mellitus in six patients (12.0%). The highest age-group burden was observed in the 61-70-year category (18/50, 36.0%; χ² = 13.00, p = 0.011). Doppler identified 76 plaque-positive vessel observations across eight evaluated vessel segments, with type II plaque being most frequent (32/76, 42.1%; p < 0.001). CTA plaque assessment showed an irregular plaque surface in 40/74 plaque observations (54.1%) and fatty or mixed plaques in 30/74 observations each (40.5%). Across five vessel segments and 250 vessel observations per modality, clinically significant stenosis (≥50% stenosis or occlusion) was noted in 14 observations (5.6%) by Doppler, 14 (5.6%) by CTA, and 18 (7.2%) by MRA; the aggregate between-modality difference was not statistically significant (χ² = 0.74, p = 0.690). Matched vessel-category stenosis percentages showed very strong aggregate correlations between Doppler and CTA (Pearson r = 0.981), Doppler and MRA (r = 0.988), and CTA and MRA (r = 0.970; all p < 0.001).

Conclusions

Color Doppler ultrasound demonstrated useful screening capability for extracranial carotid disease in ischemic stroke patients, while CTA and MRA added anatomical clarification of plaque morphology, stenosis, occlusion, and vertebrobasilar variants. The stenosis-grade distribution appeared comparable across modalities at an aggregate level; however, patient-level agreement, diagnostic accuracy measures, and outcome correlation could not be established without larger datasets containing paired raw imaging results and standardized reference criteria.

## Introduction

Stroke remains a major global cause of death and disability. Contemporary global burden estimates show that stroke continues to impose a large health and socioeconomic burden, especially in low- and middle-income settings, where access to rapid vascular imaging and secondary prevention services may be variable [1]. Ischemic stroke accounts for the majority of stroke events and is commonly related to a combination of age, hypertension, diabetes mellitus, tobacco exposure, dyslipidemia, cardioembolism, and extracranial or intracranial atherosclerotic disease [2,3]. Among the potentially treatable causes of recurrent ischemic events, extracranial carotid artery stenosis is clinically important because accurate detection may influence antiplatelet therapy, statin therapy, risk-factor control, and referral for carotid endarterectomy or stenting in selected patients [3,4].

The modern imaging evaluation of carotid and vertebral artery (VA) disease is no longer restricted to luminal narrowing alone. Although the percentage of stenosis remains central to treatment decisions, plaque morphology and vulnerability features such as irregular surface, ulceration, lipid-rich necrotic core, intraplaque hemorrhage, and calcification are increasingly recognized as relevant markers of embolic potential [5-9]. Therefore, radiology reports for stroke patients should ideally address both the degree of stenosis and the morphological features of plaques, while also identifying occlusion, hypoplastic vertebral arteries, and other vascular variants that may affect posterior circulation hemodynamics [10].

Color Doppler ultrasound is often used as an initial test because it is noninvasive, relatively inexpensive, portable, repeatable, and free from ionizing radiation. It provides real-time assessment of plaque echogenicity, luminal narrowing, peak systolic velocity (PSV), end-diastolic velocity (EDV), and spectral broadening. However, Doppler ultrasound is operator-dependent and can be limited by patient habitus, high carotid bifurcation, heavy calcification, short neck, vessel tortuosity, and difficulty in visualizing VA origins [11,12]. CT angiography (CTA) provides rapid vascular mapping with high spatial resolution and is particularly useful in acute stroke pathways and preintervention planning, but it requires iodinated contrast and exposes the patient to ionizing radiation [12]. MR angiography (MRA) can evaluate vessel patency and stenosis without ionizing radiation and may be valuable in patients with contraindications to iodinated contrast, although it may be affected by motion artifacts, flow-related artifacts, access limitations, and longer acquisition time [6,8,11].

Studies comparing Doppler ultrasound with CTA or MRA have shown variable concordance, particularly in moderate stenosis categories and VA assessment [11,12]. In practical clinical settings, especially in resource-limited hospitals, physicians often use a staged imaging approach in which Doppler acts as the initial screening tool and CTA or MRA is added for equivocal cases, high-grade stenosis, suspected occlusion, posterior circulation symptoms, or procedural planning. There remains value in documenting real-world multimodality findings among ischemic stroke patients, particularly when plaque type, CTA morphology, MRA vascular findings, and risk-factor profiles are assessed together. In this context, the objective of this study was to describe extracranial carotid and VA abnormalities in ischemic stroke patients using color Doppler ultrasound, CTA, and MRA and to assess aggregate distributional patterns of plaque characteristics and stenosis grades across these imaging modalities.

## Materials and methods

Study design and setting

This was a prospective observational imaging correlation study conducted in the Department of General Medicine, Dr. N. D. Desai Faculty of Medical Science and Research, Nadiad, Gujarat, India. The study period was one year, from December 1, 2024, to November 30, 2025. The study was approved by the Institutional Ethics Committee (NDDFMSR/IEC/28/2024).

Sample size calculation

The sample size was calculated to estimate the proportion of ischemic stroke patients with extracranial carotid or vertebral arterial abnormality on vascular imaging. Since no fixed prior local estimate was available, the anticipated proportion was taken as 50% to obtain the maximum sample size.

Using the following single-proportion formula:

$n = \frac{Z^2pq}{d^2},$

with Z = 1.96 at a 95% confidence level, p = 0.50, q = 0.50, and an absolute precision of 14%, the calculated sample size was 49.00, which was rounded to 50 patients.

Therefore, 50 patients were included in the study. The intermodality comparison of Doppler ultrasound, CTA, and MRA was exploratory and based on aggregate imaging distributions; the study was not powered for patient-level diagnostic accuracy or agreement analysis.

Study population

A total of 50 patients were included. Eligible patients had a clinical history and examination findings suggestive of stroke and had ischemic stroke confirmed on neuroimaging. Demographic data and vascular risk factors, including hypertension, diabetes mellitus, smoking, and a history of ischemic heart disease or myocardial infarction, were documented. Patients with hemorrhagic stroke, rheumatic heart disease or intracardiac clots, vasculitis, connective tissue disorders, preexisting statin therapy, head injury, or brain tumor were excluded.

Imaging protocol

All included patients underwent color Doppler ultrasound, CTA, and MRA evaluation of the extracranial carotid and vertebral arteries as clinically feasible during the same diagnostic episode. Color Doppler ultrasound was performed using a SonoScape P20 ultrasound system (SonoScape Medical Corp., Shenzhen, China). The common carotid artery (CCA), internal carotid artery (ICA), external carotid artery (ECA), and VA were assessed bilaterally. Parameters included plaque presence and type, PSV, EDV, ICA/CCA velocity ratios, spectral broadening, and degree of stenosis. Doppler angle correction was maintained at approximately 60 degrees.

CTA was performed using a GE BrightSpeed 16-slice CT scanner (GE Healthcare, Waukesha, USA). The extracranial CCA, ICA, ECA, and VA were evaluated for vessel involvement, plaque surface morphology, plaque composition, stenosis grade, and associated brain findings. MRA was performed using a Philips Achieva MR scanner (Philips Medical Systems Nederland B.V., Best, The Netherlands). Noncontrast angiographic sequences were used for extracranial vessel assessment, with gadolinium contrast administered when clinically required. MRA findings included vessel involvement, integrity, plaque characterization, stenosis grade, occlusion, and hypoplastic or thrombosed intracranial or vertebrobasilar vessels.

All 50 included patients underwent color Doppler ultrasound, CTA, and MRA during the same index hospital admission. Color Doppler ultrasound was generally performed within the first 24 hours of admission as the initial extracranial vascular screening investigation. CTA was performed within approximately 24-48 hours, and MRA was completed within approximately 48-72 hours of admission, depending on neurological stability, renal function status, contrast eligibility, and scanner availability. Thus, all three vascular imaging assessments were completed within an approximate 72-hour diagnostic window.

Operational definitions

Stenosis was categorized as 16-49%, 50-69%, 70-99%, or 100% occlusion according to the tabulated results available for Doppler, CTA, and MRA. Clinically significant stenosis for aggregate comparison was defined as ≥50% stenosis or occlusion. The degree of stenosis on color Doppler ultrasound was assessed using grayscale plaque visualization, color-flow luminal narrowing, spectral broadening, PSV, EDV, and ICA/CCA velocity ratios. For CTA and MRA, stenosis grading was performed using a NASCET-based luminal measurement approach, in which the narrowest residual lumen at the stenotic segment was compared with the distal normal reference arterial lumen. Stenosis was categorized as no stenosis, mild stenosis (16-49%), moderate stenosis (50-69%), severe stenosis (70-99%), or complete occlusion (100%). For CCA, ECA, and VA segments, luminal narrowing was assessed relative to the nearest visually normal adjacent arterial segment. Doppler plaque types were analyzed as Type I to Type V as recorded in the original study dataset. CTA plaque surface was categorized as irregular, smooth, or ulcerated, and plaque composition was categorized as fatty, mixed, or calcified.

Statistical analysis

Data were collected prospectively from the same ischemic stroke cohort using Doppler ultrasound, CTA, and MRA findings. However, only summarized aggregate vessel-level data were available for the present analysis; therefore, intermodality analysis was limited to descriptive aggregate comparison. Continuous variables were summarized as mean ± SD and 95% CI. Categorical variables were summarized as frequency and percentage. Chi-square tests were used to describe whether age-group and plaque-type distributions differed from equal distribution across categories. Aggregate modality-wise stenosis distributions were compared using chi-square tests. As an exploratory aggregate analysis, Pearson correlations were calculated between matched vessel-category stenosis percentages across modalities. A p-value <0.05 was considered statistically significant. IBM SPSS Statistics for Windows, version 24.0 (released 2016; IBM Corp., Armonk, NY, USA) was used for analysis.

## Results

Baseline demographic and risk factor profile

The study included 50 patients with ischemic stroke. The mean age was 63.6 ± 13.34 years, with a 95% CI of 59.8-67.4 years. The largest age category was 61-70 years, which included 18 patients (36.00%), followed by 51-60 years in 12 patients (24.00%) and 71-80 years in 11 patients (22.00%). There were 30 males (60.00%) and 20 females (40.00%); the male predominance was not statistically significant (p = 0.157). Hypertension and smoking were the most common risk factors, each documented in 27 patients (54.00%), while diabetes mellitus was present in six patients (12.00%). No patient had a documented history of ischemic heart disease or myocardial infarction. The results are shown in Table 1.

**Table 1:** Baseline demographic profile and vascular risk factors (n = 50). Continuous variables are presented as mean ± SD, and categorical variables are presented as n (%). A p-value <0.05 was considered statistically significant. ^NS^ indicates nonsignificance (p > 0.05).

Variable	Values	95% CI	Statistics	p-value
Age, years	63.60 ± 13.34	59.80-67.40	NA	NA
Age group in years, n (%)				
<50 years	5 (10.00)	4.30-21.40	NA	NA
51-60 years	12 (24.00)	14.30-37.40		
61-70 years	18 (36.00)	24.10-49.90		
71-80 years	11 (22.00)	12.80-35.20		
>80 years	4 (8.00)	3.20-18.80		
Gender distribution, n (%)				
Male	30 (60.00)	46.20-72.40	χ² = 2.00	p = 0.157^NS^
Female	20 (40.00)	27.60-53.80		
Vascular risk factors, n (%)				
Hypertension	27 (54.0)	40.40-67.00	NA	
Diabetes mellitus	6 (12.00)	5.60-23.80		
Smoking	27 (54.00)	40.40-67.00		

Doppler plaque type and velocity-ratio findings

Across the eight evaluated extracranial vessel segments, Doppler detected 76 plaque-positive vessel observations. Type II plaque was the most frequent Doppler plaque type (32/76, 42.11%), followed by Type III plaque (20/76, 26.32%) and Type V plaque (14/76, 18.42%). The distribution of Doppler plaque type differed significantly across plaque categories (p < 0.001). The right CCA had the highest plaque burden, with plaque in 28/50 vessel observations (56.00%), followed by the right ICA in 18/50 (36.00%) and left CCA in 18/50 (36.00%). No plaque was recorded in the right VA, left ECA, or left VA. The results are shown in Table 2.

**Table 2:** Doppler plaque type distribution according to vessel segment. Doppler plaque findings are presented as n (%) using the 50-patient denominator for each vessel segment. ^*^ indicates statistical significance (p < 0.05). CCA, common carotid artery; ECA, external carotid artery; ICA, internal carotid artery; VA, vertebral artery

Vessel	No plaque	Type I	Type II	Type III	Type IV	Type V	Statistics	p-value
Right CCA	22 (44.00)	0 (0.00)	14 (28.00)	8 (16.00)	0 (0.00)	6 (12.00)	NA	NA
Right ICA	32 (64.00)	0 (0.00)	6 (12.00)	10 (20.00)	0 (0.00)	2 (4.00)		
Right ECA	48 (96.00)	0 (0.00)	0 (0.00)	0 (0.00)	0 (0.00)	2 (4.00)		
Right VA	50 (100.00)	0 (0.00)	0 (0.00)	0 (0.00)	0 (0.00)	0 (0.00)		
Left CCA	32 (64.00)	4 (8.00)	8 (16.00)	2 (4.00)	2 (4.00)	2 (4.00)		
Left ICA	42 (84.00)	4 (8.00)	4 (8.00)	0 (0.00)	0 (0.00)	0 (0.00)		
Left ECA	50 (100.00)	0 (0.00)	0 (0.00)	0 (0.00)	0 (0.00)	0 (0.00)		
Left VA	50 (100.00)	0 (0.00)	0 (0.00)	0 (0.00)	0 (0.00)	0 (0.00)		
Overall plaque-positive observations	-	8 (10.53)	32 (42.11)	20 (26.32)	4 (5.26)	14 (18.42)	χ² = 36.54	p < 0.001^*^

Table 3 summarizes the Doppler-based spectral broadening and ICA/CCA velocity ratio findings. Spectral broadening was most commonly observed in the right CCA (10 patients (20.00%)), followed by the right ICA (6 patients (12.00%)) and left ICA (2 patients (4.00%)). The PSV ICA/CCA ratio was <1.8 in most patients, including 44 patients (88.00%) on the right side and all 50 patients (100.00%) on the left side, while a ratio >1.8 was seen only on the right side in six patients (12.00%). The EDV ICA/CCA ratio was <2.4 in 46 patients (92.00%) on both sides, whereas an elevated EDV ratio >2.4 was recorded in two right-sided examinations (4.00%) and none on the left side. EDV ratio assessment was not available or not assessable in two right-sided and four left-sided examinations.

**Table 3:** Color Doppler assessment of spectral broadening and ICA/CCA PSV and EDV ratios. CCA, common carotid artery; EDV, end-diastolic velocity; ICA, internal carotid artery; PSV, peak systolic velocity

Parameter	n (%)	95% CI
Spectral broadening, n (%)		
Right CCA	10 (20.00)	11.20-33.00
Right ICA	6 (12.00)	5.60-23.80
Left ICA	2 (4.00)	1.10-13.50
PSV ICA/CCA ratio <1.8, n (%)		
Right	44 (88.00)	76.20-94.40
Left	50 (100.00)	92.90-100.00
PSV ICA/CCA ratio >1.8, n (%)		
Right	6 (12.00)	5.60-23.80
Left	0 (0.00)	0.00-7.10
EDV ICA/CCA ratio <2.4, n (%)		
Right	46 (92.00)	81.20-96.80
Left	46 (92.00)	81.20-96.80
EDV ICA/CCA ratio >2.4, n (%)		
Right	2 (4.00)	1.10-13.50
Left	0 (0.00)	0.00-7.10
EDV ICA/CCA ratio not available/not assessable, n (%)		
Right	2 (4.00)	1.10-13.50
Left	4 (8.00)	3.20-18.80

CTA detected plaque morphology across 74 plaque observations in the five carotid/ECA vessel segments represented in the tabulated data. Irregular plaque surface was the most frequent surface pattern, observed in 40/74 plaque observations (54.1%), followed by smooth surface in 22/74 (29.73%) and ulcerated surface in 12/74 (16.22%). The distribution of CTA surface morphology was statistically uneven (p < 0.001). Fatty and mixed plaques were each observed in 30/74 observations (40.54%), while calcified plaques were documented in 14/74 (18.92%); the composition distribution was also statistically uneven (p = 0.031). The results are shown in Table 4.

**Table 4:** CTA plaque surface and composition findings according to vessel segment. CTA plaque findings are presented as n (%) using the 50-patient denominator for each vessel segment. Overall surface morphology and overall plaque composition were analyzed separately using the chi-square test. ^*^ indicates statistical significance. CCA, common carotid artery; CTA, CT angiography; ECA, external carotid artery; ICA, internal carotid artery

Vessel	Irregular	Smooth	Ulcerated	Fatty	Mixed	Calcified	Statistics	p-value
Right CCA	16 (32.00)	10 (20.00)	2 (4.00)	10 (20.00)	12 (24.00)	6 (12.00)	Descriptive	-
Right ICA	14 (28.00)	2 (4.00)	2 (4.00)	4 (8.00)	12 (24.00)	2 (4.00)	Descriptive	-
Right ECA	2 (4.00)	0 (0.00)	0 (0.00)	0 (0.00)	0 (0.00)	2 (4.00)	Descriptive	-
Left CCA	8 (16.00)	6 (12.00)	4 (8.00)	10 (20.00)	4 (8.00)	4 (8.00)	Descriptive	-
Left ICA	0 (0.00)	4 (8.00)	4 (8.00)	6 (12.00)	2 (4.00)	0 (0.00)	Descriptive	-
Overall surface morphology	40 (54.05)	22 (29.73)	12 (16.22)	-	-	-	χ² = 16.32	p < 0.001^*^
Overall plaque composition	-	-	-	30 (40.54)	30 (40.54)	14 (18.92)	χ² = 6.92	p = 0.031^*^

Stenosis was identified by all three imaging modalities in the right CCA, right ICA, right ECA, left CCA, and left ICA vessel segments. Mild stenosis (16-49%) formed the largest category across all modalities. High-grade stenosis (70-99%) and occlusion were less frequent but were consistently captured in key carotid segments. On aggregate vessel-observation analysis, clinically significant stenosis or occlusion (≥50%) was observed in 14/250 observations (5.6%) by Doppler, 14/250 (5.6%) by CTA, and 18/250 (7.2%) by MRA. The aggregate difference between modalities for clinically significant stenosis was not statistically significant (p = 0.69). The results are shown in Table 5.

**Table 5:** Stenosis grading by vessel segment and imaging modality. Stenosis grades are presented as n (%) for each vessel segment and imaging modality using the 50-patient denominator. Vessel-level entries are descriptive because patient-level paired cross-tabulations were not available. CCA, common carotid artery; CTA, CT angiography; ECA, external carotid artery; ICA, internal carotid artery; MRA, MR angiography

Vessel	Modality	Stenosis grading, n (%)			
		16-49%	50-69%	70-99%	100% occlusion
Right CCA	Doppler	18 (36.00)	4 (8.00)	2 (4.00)	0 (0.00)
Right CCA	CTA	20 (40.00)	2 (4.00)	2 (4.00)	0 (0.00)
Right CCA	MRA	18 (36.00)	6 (12.00)	2 (4.00)	0 (0.00)
Right ICA	Doppler	12 (24.00)	0 (0.00)	2 (4.00)	4 (8.00)
Right ICA	CTA	10 (20.00)	0 (0.00)	2 (4.00)	6 (12.00)
Right ICA	MRA	10 (20.00)	0 (0.00)	2 (4.00)	6 (12.00)
Right ECA	Doppler	2 (4.00)	0 (0.00)	0 (0.00)	0 (0.00)
Right ECA	CTA	2 (4.00)	0 (0.00)	0 (0.00)	0 (0.00)
Right ECA	MRA	2 (4.00)	0 (0.00)	0 (0.00)	0 (0.00)
Left CCA	Doppler	18 (36.00)	0 (0.00)	0 (0.00)	2 (4.00)
Left CCA	CTA	16 (32.00)	0 (0.00)	0 (0.00)	2 (4.00)
Left CCA	MRA	18 (36.00)	0 (0.00)	0 (0.00)	2 (4.00)
Left ICA	Doppler	4 (8.00)	0 (0.00)	0 (0.00)	0 (0.00)
Left ICA	CTA	2 (4.00)	0 (0.00)	0 (0.00)	0 (0.00)
Left ICA	MRA	6 (12.00)	0 (0.00)	0 (0.00)	0 (0.00)

Table 6 presents the aggregate stenosis distribution across five vessel segments, with 250 vessel observations assessed for each imaging modality. No stenosis was the most common finding across Doppler, CTA, and MRA, followed by mild stenosis of 16-49%. Clinically significant stenosis, defined as ≥50% stenosis or complete occlusion, was observed in 14 observations (5.60%) each on Doppler and CTA and in 18 observations (7.20%) on MRA. The overall stenosis-grade distribution did not differ significantly among the three modalities (p = 0.949), and the comparison of clinically significant stenosis was also nonsignificant (p = 0.690).

**Table 6:** Aggregate stenosis distribution across five vessel segments and 250 vessel observations per modality. Aggregate values were calculated across five vessel segments and 250 vessel observations for each modality. Clinically significant stenosis was defined as ≥50% stenosis or complete occlusion. χ², chi-square statistics comparing aggregate stenosis-category distribution and clinically significant stenosis across modalities. ^NS^ indicates nonsignificance (p > 0.05). CTA, CT angiography; MRA, MR angiography

Modality	No stenosis	16-49%	50-69%	70-99%	100% occlusion	Clinically significant (≥50%)	Statistics	p-value
Doppler	182 (72.80)	54 (21.60)	4 (1.60)	4 (1.60)	6 (2.40)	14 (5.60)	-	-
CTA	186 (74.40)	50 (20.00)	2 (0.80)	4 (1.60)	8 (3.20)	14 (5.60)	-	-
MRA	178 (71.20)	54 (21.60)	6 (2.40)	4 (1.60)	8 (3.20)	18 (7.20)	-	-
Overall stenosis-category comparison	-	-	-	-	-	-	χ² = 2.74	p = 0.94^NS^
Clinically significant stenosis comparison	-	-	-	-	-	-	χ² = 0.74	p = 0.690^NS^

Doppler vs CTA Pearson r = 0.981 (p < 0.001), Doppler vs MRA r = 0.988 (p < 0.001), and CTA vs MRA r = 0.970 (p < 0.001) (Table 7). These findings reflect only aggregate distributional similarity across modalities and should not be interpreted as diagnostic equivalence, interchangeability, or patient-level agreement because individual paired vessel-level classifications were not available. The Pearson correlations were calculated using 20 matched aggregate data points derived from five vessel segments across four stenosis categories; therefore, these values reflect similarity in aggregate percentage distributions only and do not indicate patient-level or vessel-level agreement between modalities.

**Table 7:** Exploratory aggregate distributional correlation across imaging modalities. Pearson correlation r values describe distributional similarity, while χ² is the chi-square value. ^*^ indicates statistical significance. Correlation coefficients were calculated from aggregate vessel-category percentages using 20 matched data points across five vessel segments and four stenosis categories. These exploratory correlations describe distributional similarity only and should not be interpreted as patient-level diagnostic agreement, concordance, equivalence, or interchangeability between imaging modalities. CTA, CT angiography; MRA, MR angiography

Comparison	Statistics	p-value
Doppler vs CTA	Pearson r = 0.981	p < 0.001^*^
Doppler vs MRA	Pearson r = 0.988	p < 0.001^*^
CTA vs MRA	Pearson r = 0.970	p < 0.001^*^

MRA and CT/MR brain angiographic findings

In CT/MR brain angiographic analysis, no abnormality was detected in 14 patients (28.0%). The most common abnormality was involvement of the M2 part of the right MCA, observed in 10 patients (20.0%). A1 segment involvement of the left ACA, left PCA involvement, M2 part of the left MCA, and right VA hypoplasia with PCA involvement were each observed in four patients (8.0%). MRA also documented hypoplasia of the right VA in four patients (8.0%) and left VA in two patients (4.0%) in the available results (Table 8).

**Table 8:** CTA/MRA distribution of thrombosed or hypoplastic vessels. CTA/MRA findings are presented as n (%) among 50 patients. ACA, anterior cerebral artery; CTA, CT angiography; MCA, middle cerebral artery; MRA, MR angiography; PCA, posterior cerebral artery; VA, vertebral artery

Findings	n (%)	95% CI
A1 part of left ACA	4 (8.00)	3.20-18.80
A1 part of right ACA	2 (4.00)	1.10-13.50
Left PCA	4 (8.00)	3.20-18.80
Left VA hypoplastic	2 (4.00)	1.10-13.50
M2 part of left MCA	4 (8.00)	3.20-18.80
M2 part of right MCA	10 (20.00)	11.20-33.00
M2 segment of left MCA	2 (4.00)	1.10-13.50
M3 part of left MCA	2 (4.00)	1.10-13.50
M3 part of right MCA	2 (4.00)	1.10-13.50
Right VA hypoplastic with PCA involvement	4 (8.00)	3.20-18.80
No abnormality detected	14 (28.00)	17.50-41.70

## Discussion

The present study evaluated color Doppler ultrasound, CTA, and MRA findings in 50 patients with ischemic stroke and demonstrated a clinically relevant pattern of extracranial vascular disease. The cohort was dominated by older adults; males formed 60% of cases, hypertension and smoking were the most frequently recorded risk factors, Doppler most commonly identified Type II plaques, and CTA most commonly demonstrated irregular plaque morphology. These findings support the practical value of comparing noninvasive modalities in stroke patients, similar to recent work showing that Doppler and CTA may provide complementary information in the evaluation of cervical arterial stenosis [13].

The plaque findings in this study are clinically important because carotid atherosclerosis is no longer interpreted only by the percentage of luminal narrowing. Surface irregularity, ulceration, echogenicity, calcification, lipid-rich components, and mixed plaque morphology can influence embolic potential and may explain stroke events even in patients without very high-grade stenosis. The present CTA-based morphology table therefore strengthens the manuscript by showing plaque surface and composition, which is consistent with recent evidence emphasizing the role of ultrasound and CT in identifying plaque vulnerability [14].

CTA contributed important anatomical detail by demonstrating plaque type, surface morphology, stenosis category, and intracranial or cervical vessel involvement. Its strength in acute and subacute stroke imaging is its speed, spatial resolution, wide availability, and ability to evaluate the arterial tree from the aortic arch to intracranial branches in a single acquisition. In the present dataset, CTA was particularly useful for plaque characterization and for confirming severe stenosis or occlusion, which aligns with contemporary descriptions of vascular CTA as a rapid and informative technique for arterial disease assessment [15].

Color Doppler ultrasound remained valuable as the initial screening modality because it is noninvasive, inexpensive, repeatable, and capable of assessing flow velocity, spectral broadening, plaque echogenicity, and hemodynamic significance. In this study, the right CCA and right ICA showed notable Doppler abnormalities, while the ICA/CCA velocity ratio was elevated in a smaller subgroup. The practical interpretation is that Doppler is useful for triage and surveillance, while confirmatory CTA or MRA may be required when Doppler suggests clinically significant stenosis or when the anatomy is difficult to visualize [16].

The additional aggregate statistical analysis showed no significant difference in the overall distribution of stenosis categories among Doppler, CTA, and MRA. Although the overall frequencies were similar, this does not establish that the modalities are equivalent or interchangeable, as individual patients may still have been classified differently by each modality. However, aggregate similarity does not prove patient-level agreement because two modalities may produce similar totals while classifying individual patients differently. For this reason, the revised manuscript correctly avoids overclaiming diagnostic accuracy and recommends that patient-wise paired raw data be used in future revisions to calculate kappa agreement, sensitivity, specificity, predictive values, and ROC curves [17].

The degree of carotid stenosis remains an important marker of future stroke risk, but recent evidence indicates that risk assessment should not be restricted to stenosis severity alone. In the present study, most vessel observations were in the lower stenosis categories, while severe stenosis and occlusion were less frequent. This distribution is clinically plausible because many ischemic stroke patients may have vulnerable plaque, embolic sources, or intracranial pathology without uniformly severe extracranial narrowing [18].

The CTA observation of irregular and ulcerated plaque is relevant because such features are commonly considered markers of higher-risk atherosclerotic disease. In clinical practice, these findings can influence the urgency of neurological, vascular, and medical risk-reduction decisions even when the measured stenosis is not extreme. Therefore, the revised discussion emphasizes that plaque morphology should be reported together with stenosis grade rather than being treated as a secondary or incidental finding [19].

The comparison of Doppler, CTA, and MRA in this study also highlights the need for modality-specific interpretation. Doppler provides functional hemodynamic information, CTA provides high-resolution luminal and calcific plaque detail, and MRA can demonstrate vessel integrity without ionizing radiation or iodinated contrast. A balanced multimodality pathway is therefore more clinically useful than presenting one modality as universally superior for every patient and every vascular segment [20].

Carotid imaging has evolved from a simple stenosis-measurement approach to a broader assessment of plaque biology, vascular anatomy, surface morphology, and patient-level risk. This is particularly important for ischemic stroke patients, where the responsible mechanism may include artery-to-artery embolism, small-vessel disease, cardioembolism, vertebrobasilar disease, or mixed pathology. The present manuscript has been revised to reflect this broader interpretation by linking the imaging findings with clinical risk factors and infarct-related vascular observations [21].

The clinical profile of the present cohort is consistent with the usual vascular risk background of ischemic stroke. Hypertension and smoking were the most frequent documented risk factors, and both can promote endothelial dysfunction, arterial stiffness, plaque progression, and thrombosis. Diabetes was recorded in a smaller proportion of patients, and myocardial infarction was not documented in this dataset; however, these findings should be interpreted cautiously because the sample size was limited and the available dataset did not permit multivariable adjustment [22].

From a stroke mechanism perspective, extracranial carotid and VA imaging is most useful when interpreted alongside the clinical presentation, infarct territory, cardiac evaluation, and risk-factor profile. In the present study, CT and MR angiographic findings included intracranial vessel abnormalities and VA hypoplasia, showing that vascular evaluation should not stop at the carotid bifurcation alone. This supports the revised framing of the paper as an imaging correlation study in confirmed ischemic stroke rather than a purely carotid stenosis study [23].

Future studies may improve plaque characterization by adding contrast-enhanced ultrasound, particularly for identifying intraplaque neovascularization and surface activity. Although this technique was not used in the present work, it is relevant because the current dataset already demonstrates plaque heterogeneity across Doppler and CTA. Adding contrast-enhanced ultrasound in a larger cohort may help determine whether apparently moderate plaques have biologically high-risk characteristics [24].

High-resolution MRI is another important method for evaluating vulnerable plaque components such as lipid-rich necrotic core, intraplaque hemorrhage, fibrous cap status, and mural thrombus. The present study used MRA primarily for vessel assessment and stenosis categorization, but future protocols could benefit from dedicated vessel-wall imaging sequences. Such an approach would make the comparison between ultrasound, CTA, and MRI more biologically meaningful rather than only luminal [25].

The VA findings deserve attention because posterior circulation stroke mechanisms may be underrecognized when imaging is focused mainly on the carotid arteries. In this cohort, VA hypoplasia was documented on MRA in a small subset of patients, and intracranial vascular abnormalities were also recorded. These findings suggest that future studies should separately analyze anterior and posterior circulation infarcts and relate them to extracranial and intracranial vascular variants [26].

A key methodological improvement for future work would be the construction of a patient-level prediction model that combines age, sex, hypertension, diabetes, smoking, Doppler plaque type, velocity ratios, CTA morphology, and MRA findings. The currently available data were summarized in aggregate form, so such modeling could not be performed. Preserving raw patient-wise data would allow logistic regression or machine-learning models to identify which clinical and imaging variables best predict clinically significant stenosis or vulnerable plaque [27].

Standardized plaque reporting would further improve the manuscript and its clinical usefulness. The current tables report Doppler plaque type and CTA morphology, but the terminology should be harmonized so that plaque surface, composition, calcification, ulceration, stenosis grade, and suspected vulnerability are described consistently. Incorporating a structured plaque-risk reporting framework in future studies may improve reproducibility and make the findings easier to compare across institutions [28-30].

Strengths and limitations

The main strength of this study is that it evaluates three commonly used noninvasive imaging modalities in the same ischemic stroke population and presents the results with corrected n (%), CIs, revised tables, clear legends, and moderated interpretation. The addition of plaque morphology is especially useful because vulnerable plaque features may be linked to recurrent ischemic events and may provide information beyond luminal narrowing alone.

The sample size was small, the study was single-center, the data available for revision were aggregate rather than patient-wise, and digital subtraction angiography was not used as an independent reference standard. Blinding between modality interpretations was not documented, Doppler is operator-dependent, and long-term outcomes such as recurrent stroke, intervention, mortality, and functional recovery were not assessed. Another limitation is that the sample size was calculated for estimating the proportion of vascular imaging abnormalities rather than for a formal patient-level agreement or diagnostic accuracy comparison between Doppler ultrasound, CTA, and MRA. In addition, multiple vessel segments were assessed from the same patients, and within-patient clustering could not be adjusted without patient-wise paired vessel-level data; therefore, the aggregate chi-square findings should be interpreted cautiously. Future studies should use standardized raw-data collection so that imaging-defined plaque stability can be tested against clinical stroke risk more robustly.

## Conclusions

In this prospective observational imaging correlation study of 50 ischemic stroke patients, older age, male sex, hypertension, and smoking were common clinical features. Color Doppler ultrasound demonstrated carotid plaque and hemodynamic changes, with Type II plaque being the most frequent Doppler plaque category. CTA provided additional information on plaque surface and composition, with irregular plaque surface being the most common CTA morphology. MRA further contributed to the assessment of vessel integrity, occlusion, and vertebrobasilar variants. Although aggregate stenosis distributions appeared similar across Doppler ultrasound, CTA, and MRA, the available data do not permit conclusions regarding diagnostic superiority, screening performance, interchangeability, or patient-level agreement. Larger studies using paired patient-level imaging data, standardized reference criteria, and outcome correlation are required to define the comparative diagnostic value of these modalities in ischemic stroke evaluation.
